# Association between enjoyment, physical activity, and physical literacy among college students: a mediation analysis

**DOI:** 10.3389/fpubh.2023.1156160

**Published:** 2023-06-15

**Authors:** Wenjing Yan, Leqin Chen, Lina Wang, Yihan Meng, Ting Zhang, Hongjuan Li

**Affiliations:** ^1^School of Sport Science and Key Laboratory of the Ministry of Education of Exercise and Physical Fitness, Beijing Sport University, Beijing, China; ^2^School of Physical Education, Shanxi Normal University, Taiyuan, Shanxi, China

**Keywords:** enjoyment, physical literacy, physical activity, college students, mediation

## Abstract

**Background:**

Physical literacy and enjoyment are important factors that affect physical activity.

**Purpose:**

This work studies whether physical activity enjoyment (PAE) mediates the association between moderate to vigorous physical activity (MVPA) and physical literacy (PL) among college students.

**Methods:**

Chinese college students were recruited using the Perceived Physical Literacy Instrument Scale (PPLI-SC), the International Physical Activity Questionnaire Short Form (IPAQ-SF), and the Physical Activity Enjoyment Scale. The SPSS Hayes process macro (model 4) was used to analyze the direct impact and the indirect impact. Pearson correlation, independent sample *t*-tests, and linear regression were used to analyze the relationship between indicators.

**Results:**

The study surveyed 587 boys and 1,393 girls with a total of 1,980 valid questionnaires. MVPA, PAE, and PL of boys were significantly higher than girls (*p* < 0.01). The correlation analysis showed that MVPA, PL, and PAE were significantly correlated (*p* < 0.01). The results showed the direct effect of PL on MVPA was still statistically significant (β = 0.067, *p* < 0.05) after adding PAE variables; PAE has a positive effect on MVPA after controlling PL (β = 0.170, *p* < 0. 01). PL has a positive effect on PAE (β = 0.750, *p* < 0.01). PL impacted MVPA as explained by a 65.58% mediating effect of enjoyment.

**Conclusion:**

Physical activity enjoyment mediates the relationship between PL and MVPA among college students. This means that even high PL among student may not imply that they are physically active if they do not enjoy physical activity.

## 1. Introduction

Physical activity (PA) is defined as any bodily movement produced by skeletal muscles that requires energy expenditure (1). PA can produce a range of health benefits including physical and mental health (2). Greater amounts and higher intensities of PA as well as different types of physical activity (aerobic and muscle and bone strengthening activities) are associated with improved health outcomes (3). Exercise can resolve the positive symptoms of depression and increase pleasure (4, 5). Vigorous physical activity (VPA) is particularly important for health because it is associated with lower body fat and better cardiorespiratory health vs. low-intensity PA (6). Moderate-to-vigorous physical activity (MVPA) has the greatest benefit in prolonging lifespan (7). Increases in sedentary behavior significantly elevate stress, anxiety, and depression among college students (8, 9). A lifestyle with sedentary and low-level PA has evolved into a public health problem (10). Significant changes have occurred in terms of sports activities among college students (11).

Short-term health intervention can help college students to establish a healthy lifestyle with positive impacts on their healthy behaviors (12). Recently, the concept of physical literacy (PL) has attracted increasing research attention in physical education, sports participation and the promotion of PA. In 2007, Whitehead optimized the concept of PL for the first time believing that PL is the motivation, confidence, physical ability, knowledge, and understanding of individuals to maintain their physical activities at an appropriate level throughout their lives (13). The International Physical Literacy Association (IPLA) was founded in 2013 under the chairmanship of Whitehead and defined the concept of “PL.” Here, PL is defined as the motivation, confidence, physical ability, knowledge, and understanding that people need to attach importance to, and take part in physical activities in their life. PL can promote individual PA, and thus it is necessary to use PL assessment tools to help understand people’s PL levels (14). Although the concept of PL has been continuously optimized and improved, Whitehead retained elements like motivation, confidence, physical ability, knowledge, and understanding. This work highlights the personal characteristics of PL and emphasizes the concept of PL as applicable to the entire life cycle. This reflects its lifelong characteristics. PL not only focuses on sports skills and PA, but also on emotional and motivational fields such as ability and self-confidence. The intersection of sports skills, positive emotions, and motivation is the core element required to ensure active activities—these are all crucial to maintaining physical activities over the life span. There are many assessment tools for PL such as the Canadian Assessment for Physical Literacy (CAPL) (15), the Physical Literacy Assessment for Youth (PLAY) (16), and the Perceived Physical Literacy Instrument (PPLI) (17). Of these, the PPLI has the most extensive attributes with multiple measurement dimensions: motivation, confidence, physical ability, and interaction with the environment. The relevant population is college students (17).

Physical activity and PL are determinants of health, and PA level can be increased by intervening PL. People with higher PL levels will have more confidence and ability to participate in various sports activities. In contrast, they will also have less PA (18). Physical activity enjoyment (PAE) is another important factor affecting sports continuity (19). Research shows that subjective emotional responses to PA and enjoyment are powerful predictors of continuous participation in PA (20). Researchers determined that childhood memories of enjoyment and non-enjoyment during PE are linked with attitudes toward PA, intention, and sedentary behavior into adulthood (21). While there are several forms of motivation, intrinsic motivation is distinctly important to foster PA behaviors because it promotes inherent satisfaction with an activity and is reflected in enjoyment (22). PAE is the main reason for exercisers to participate in sports activities over the long term. It is also an important factor to motivate their participation in, and adherence to, sports activities (23, 24).

However, our understanding of the association between PAE, PA, and PL is limited. It seems that PL promotes PA, and enjoyment is an important motivation to participate in PA. This study tests the hypothesis that PL increases PA through the mediation of enjoyment. Therefore, this study aims to determine whether the enjoyment of PA can modulate the relationship between PL and PA among college students.

## 2. Methods

### 2.1. Study design and participants

This is a cross-sectional study. Participants were randomly invited to fill out questionnaires in China. The selection criteria are freshmen- to junior-level college students. Students’ PL, MVPA, and PAE were measured with the Perceived Physical literacy Scale PPLI-SC (25), the International Physical Activity Questionnaire (IPAQ-S) (26), and the Physical Activity Enjoyment scale (PAE-C) (27). The study also collected demographic information (gender, age, height, and weight). There were 1980 valid questionnaires. Before answering the questionnaire, the participants had to give informed consent. This study was approved by the Scientific Experiment Ethics Committee of Beijing Sport University (2019101H). A time was from November to December 2021.

### 2.2. Physical literacy

Ma revised the original 18 questions of the PPLI-SC into eight questions suitable for Chinese college students. This eight-item instrument measured the perceived PL of Chinese undergraduates (25). Motivation, confidence, physical competition, and interaction with the environment are the dimensions of PL. This scale uses a five-point Likert scale from strongly disagreeing to strongly agreeing. The score is the sum of the numbers between 8 and 40. A higher score implies better PL.

### 2.3. Physical activity

Physical activity was investigated with the IPAQ-SF. This scale is used to evaluate the frequency and duration of light, moderate and vigorous PA as well as meditation in the past week *via* seven questions. Moderate-to-vigorous intensity refers to PA that is performed at 3.0 or more METS. The duration of the questionnaire is measured in hours and minutes, and the activity frequency is days. The responses are then translated into metabolic equivalents (MET minutes). The MVPA score refers to the sum of moderate and vigorous PA scores. The reliability test of s-IPAQ is 0.80 (26).

### 2.4. Physical activity enjoyment

We then used the enjoyment scale of physical activities to evaluate enjoyment (27). The scale consists of 16 items scored on a five-point Likert scale from 1 point (totally disagree) to 5 points (totally agree). The total score is between 16 and 80. A higher the total score implies a higher degree of enjoyment. The scale is reliable and valid (28).

### 2.5. Data analysis

**Figure 1:**
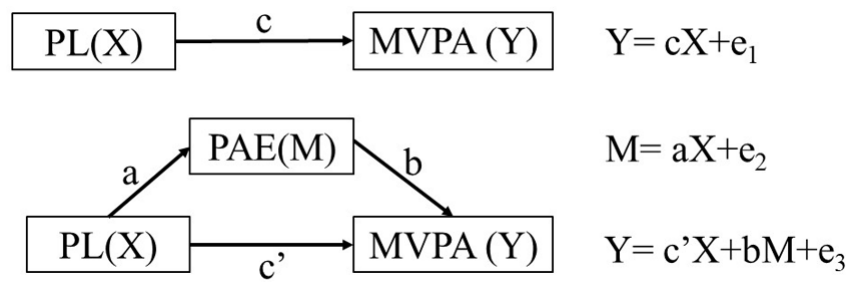
Schematic diagram of mediation effect (Hayes, A F: The Guilford Press).

The overall framework of the theory assumes that PL is the independent variable X, MVPA is the dependent variable Y, and PAE is the intermediary variable M. PL will thus positively affect MVPA, and PAE plays an intermediary role between PL and MVPA (Figure 1).

Data analyses were performed using IBM SPSS 22. The SPSS Hayes process macro (model 4) was used to analyze the direct impact and the indirect impact. The total effect is the sum of the direct effect and the indirect effect. We used Pearson correlation and linear regression to analysis the relationship between indicators. We used independent sample *t*-tests for gender difference analysis. The coefficient of variation (CV) reflected the degree of data dispersion. *p* < 0.05 was considered statistically significant.

## 3. Results

### 3.1. Student characteristics

There are 1980 effective persons in this survey including 587 males accounting for 29.6% and 1,393 females accounting for 70.4% with an average age of 20.16 ± 1.21 years (Table 1).

**Table 1:** Statistics of basic student information.

	Boy *N* = 587(29.6%)	Girl *N* = 1,393(70.4%)
Age (years)	20.33 ± 1.22	20.09 ± 1.20
High (cm)	177.64 ± 6.07	163.24 ± 5.20
Weight (kg)	71.48 ± 12.77	54.67 ± 7.98
**Grade**		
Freshman	866	43.70%
Sophomore	522	26.40%
Junior	592	29.90%
Major		0.00%
PE	383	19.40%
NO-PE	1,597	80.60%

### 3.2. Differences between boys and girls

The PL, PAE, and MVPA of boys were significantly higher than girls (*p* < 0.01). The coefficient of variation of MVPA in girls is higher than that in boys, while the coefficient of variation of PAE and PL in girls is slightly lower than that in boys. There is a significant degree of change in girls’ participation in physical activities (Table 2).

**Table 2:** Differences between boys and girls.

	Boy *N* = 587	CV	Girl *N* = 1,393	CV	T	*p*	95% CI	
Moderate to vigorous physical activity (MVPA) (met*minutes/week)	4303.45 ± 3439.21	0.80	2061.70 ± 2371.50	1.15	14.414	0.000	1936.482	2547.012
Physical activity enjoyment (PAE)	64.16 ± 14.44	0.23	57.52 ± 10.62	0.18	10.042	0.000	5.336	7.929
Physical literacy (PL)	32.01 ± 6.43	0.20	29.22 ± 5.21	0.18	9.285	0.000	2.196	3.373

### 3.3. Mediating effect test

**Figure 2:**
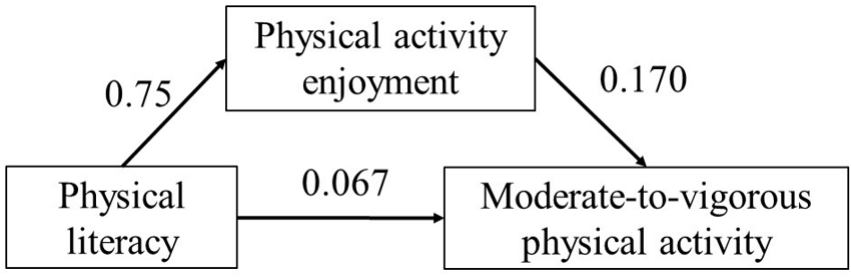
Mediation model diagram.

The results showed that there was a significant correlation between PL and MVPA (*r* = 0.195, *p* < 0.01), PL and PAE (*r* = 0.75, *p* < 0.01), as well as MVPA and PAE (*r* = 0.22, *p* < 0.01). The correlation between the study variables allowed a mediation model to be constructed to explore the mechanism of PAE in PL and MVPA. The mediation effect analysis can be used to construct three regression equations to verify the mediation effect of PAE in the relationship between PL and MVPA (Table 3). The results showed that the direct effect of PL on MVPA was still statistically significant after adding PAE variables (β = 0.067, *p* < 0.05). PAE has a positive effect on MVPA after controlling PL (β = 0.170, *p* < 0.01), and PL has a positive effect on PAE (β = 0.750, *p* < 0.01). The coefficients c, c’, a, and b were all significant, thus indicating that PAE played a partial intermediary role. Bootstrap methods were used to repeat sampling 5,000 times to test the mediation effect. The 95% confidence interval of the mediation effect of path 1 does not include 0, which indicates that the mediation effect is statistically significant and accounts for 65.58% of the effect. In summary, the results show that PL can directly affect MVPA. MVPA is also impacted via the mediation of PAE (Figure 2).

**Table 3:** Variable regression analysis and intermediary effect test.

Equation	Effect	*F*	*p*	*β*	*t*	*p*		
MVPA = cPL + e_1_	c	77.906	0.000	0.195	8.826	0.000		
PAE = aPL + e_2_	a	2541.350	0.000	0.750	50.412	0.000		
MVPA = c’PL + bPAE+e_3_	c’	52.661	0.000	0.067	2.023	0.043		
	b			0.170	5.139	0.000		
								
	Effect	BootSE	*t*	*p*	LLCI	ULCI	c’_cs	Effect
Total effect of X on Y	98.942	11.210	8.827	0.000	76.958	120.927	0.195	
Direct effect of X on Y	34.055	16.836	2.023	0.043	1.036	67.074	0.067	34.42%
Indirect effect(s) of X on Y	Effect	BootSE	BootLLCI	BootULCI				
PAE	64.888	14.723	34.671	91.687				65.58%

Adjusts gender, grade, and major.

## 4. Discussion

This study evaluated the mediating role of PAE in the relationship between PL and MVPA in college students. We found that PAE completely mediated the relation between PL and MVPA. In the strategy of promoting PA, we not only need to focus on improving the PL level of college students, but also need to let college students enjoy activities. In traditional intervention strategies for PA, the focus is on increasing PA and knowledge. The results of this study emphasize that emotional dimensions account for a large proportion of PA promotion. To improve the PA level of college students, we should not only focus on improving their PA and cognition, but also make them choose their favorite sports.

Physical literacy is the basis and prerequisite for individuals to participate in PA for life, and it is a comprehensive concept that contains three dimensions (29). Since Whitehead first proposed the PL concept, more and more countries have begun to design PL-based health promotion models to improve PA and health (30). By promoting the strategy of promoting PA and PL, students can master PL-related knowledge and skills, cultivate students’ awareness of lifelong exercise, and enhance students’ motivation to participate in PA. Raymond and colleagues measured the PL of Hong Kong students and physical education teachers using PPLI-SC. They showed that PL and PA were linearly correlated (17). Studies have shown that the intervention strategy of PL can maintain the health of freshmen and effectively reduce the decline trend of PA in freshmen (31). Interventions based on PL currently include both overall and partial interventions, with a focus on improving various dimensions of physical literacy to promote an increase in physical activity. Peter Holler found that a holistic intervention strategy could promote the physical literacy level of adults with physical activity deficiencies (32). From the improvement results of various dimensions of physical literacy, the intervention group participants showed significant improvements in physical activity behavior, confidence, and self-efficacy, but there were no changes in physical exercise attitudes, exercise knowledge, and motivation. Matthew conducted a 12 week physical literacy based game intervention on college students, and the results showed that the PL level of the intervention group of college students improved—it was mainly manifested in the improvement of confidence, knowledge, and understanding ability. There was no significant improvement effect on the dimension of sports ability (33). Therefore, further exploration is needed on strategies for promoting adult physical literacy interventions. PL intervention can only be performed in a pleasant environment, and the intervention effect of developing PL is better. Conversely, students’ confidence and motivation to actively participate in PA becomes difficult (30); this conclusion is similar to our results. Indeed, research shows that pleasure is positively correlated with intrinsic motivation. If the pleasure of physical activities is high, then the ability is high because the intrinsic motivation is high. That is, the fun of the sports activities reduces the tension and is an intrinsic motivator (34).

Our results showed that enjoyment was a very important mediating factor in the relationship between PL and MVPA. The mediation effect accounted for 65.58% of the total effect. One of the barriers for college students to participate in PA is not enjoying PA. PA is also influenced by many other factors such as self-efficacy, motivation, fear, anxiety, and depression (35, 36). Enjoyment also mediates the relationship between motivation and PA, which means that even highly active people may not engage in PA if they do not enjoy PA (37). Some studies have found that students who enjoy PA report higher levels of PA and lower levels of SB than students who enjoy PA at medium or low levels (38, 39). Enjoyment can also cushion age-related PA declined (40). The subjective enjoyment attached to PA is more important than the total amount of PA (41). We can help students determine their favorite PA and promote lifelong PA habits by providing them with more opportunities for various activities (42).

We found that the direct effect of enjoyment was 65.58%, thus indicating that the emotional dimension is more important in the intervention process of promoting physical activity based on PL. Enjoyment has the greatest impact on activity persistence. Studies have found that enjoyment not only makes PA more active but is also important for maintaining long-term persistence (43). Enjoyment in activities regulates the influence of exercise intervention on sports persistence or continuous exercise (44). Different forms of exercise have different effects on enjoyment and PA. Some studies have found that enjoyment and cohesion are important factors for persisting in regular sports and PA—these are an important results for PA intervention. Team sports seem to be more conducive to the development of enjoyment and cohesion through positive social interaction and games. Indeed, these are both factors with a positive impact on the health results of the intervention (45, 46). The enjoyment produced by different types of exercise was also different. One systematic review found that moderate-intensity continuous training sessions were enjoyment, but more enjoyment was produced after high-intensity interval training (HITT) (47). Therefore, intermittent exercise may be a viable alternative to continuous exercise (48). Some studies also found that self-efficacy might have an important impact on the enjoyment of PA. The development of a PA promotion plan should focus on improving self-efficacy belief and enjoyment of the PA experience (49).

It can be difficult to implement recommended PA and exercise guidelines; thus, it seems very important to evaluate the enjoyment of PA. Many researchers hope that physical education can provide structured MVPA, but the main goal of physical education is teaching, i.e., to impart skills, provide knowledge, and improve the ability and fun of lifelong physical activities (50). The physical health of college students is a focus of Chinese education departments. To increase college students’ PA, we should not only encourage them to improve their PL but also let them choose their favorite sports. This view is supported by the theory of self-determination, which holds that increasing the autonomy selectivity to choose to participate in the movement can build intrinsic motivation for continued participation in PA (51). Enjoyment is an important motivation in PA. When formulating intervention measures, specific types of motivation should be cultivated to improve PA, the correlation between age and gender should be considered (52). These findings require the formulation of PA-promotion plans and health-promotion interventions to encourage happy PA and improve PA enjoyment.

This study has several limitations. First, survey methods can lead to recall bias and overestimation of self-reported survey test results. Second, the proportion of females is higher than males due to the complete random collection of subjects—this may lead to some bias in the results. Third, the participants in the study are college students, with a target audience of college students. Further research is needed on whether the research methods are suitable for children and adolescents across a wider age group.

## 5. Conclusion

We found that PAE mediates the relationship between PL and MVPA among college student. This means that even students with high PL may not be physically active if they do not enjoy the PA. Girls have lower PA, PL, and PAE than boys; thus, it is necessary to give different incentives to these groups and provide different sports programs for different genders. The results found that the emotional dimension accounts for a relatively high proportion of the increase in PA. Therefore, to promote PA, the community should focus on physical ability and cognition, as well as emotional experience of the students. Future research on PL and PA promotion strategies should focus on exploring methods to improve students’ emotional health.

## Data availability statement

The original contributions presented in the study are included in the article/supplementary material, further inquiries can be directed to WY (2021yan@bsu.edu.cn).

## Ethics statement

The studies involving human participants were reviewed and approved by the Scientific Experiment Ethics Committee of Beijing Sport University (2019101H). A time was from November to December 2021. The patients/participants provided their written informed consent to participate in this study.

## Author contributions

WY, LC, LW, YM, TZ, and HL were significantly involved in the creation of the research questions and implementation of this research. WY and HL initiated the idea, carried out the analysis, and wrote the first drafted most of the article. LW and LC assisted in the analysis process. YM and TZ contributed to the content and drafted individual parts of the manuscript. All authors contributed to the article and approved the submitted version.

## Funding

This study was supported by the Fundamental Research Funds for the Central Universities (Grant Number: 2022YB010) and the Key Project for Education of the National Social Science Foundation of China (Grant number ALA190015).

## Conflict of interest

The authors declare that the research was conducted in the absence of any commercial or financial relationships that could be construed as a potential conflict of interest.

## Publisher’s note

All claims expressed in this article are solely those of the authors and do not necessarily represent those of their affiliated organizations, or those of the publisher, the editors and the reviewers. Any product that may be evaluated in this article, or claim that may be made by its manufacturer, is not guaranteed or endorsed by the publisher.
